# The Thinking Healthy Programme: Effectiveness, Scalability, and Mechanisms for Reducing Perinatal Depression Across Diverse Settings and Populations?

**DOI:** 10.1192/bjo.2026.11632

**Published:** 2026-06-30

**Authors:** Aliza Khaliq

**Affiliations:** University of Liverpool, Liverpool, United Kingdom

## Abstract

**Aims:**

Perinatal depression is a significant public health concern with far-reaching consequences for mothers, their infants, families, and society. The economic burden is substantial; in the UK, the estimated lifetime cost of perinatal depression is £75,728 per woman with an average cost of £8,500 per birth. The Thinking Healthy Programme (THP) is a community based intervention integrating cognitive and behavioural strategies into the routine work of community health workers (CHWs). An accessible, practical, and cost effective approach for low resource settings.

This scoping review aims to analyse the effectiveness of the THP in reducing depressive symptoms, improving long-term maternal and child outcomes, and assessing its cost-benefit, cultural adaptability and scalability.

**Methods:**

The methods followed the framework outlined by Arksey and O’Malley and were reported based off the Preferred Reporting Items for Systematic reviews and Meta-Analyses extension for Scoping Reviews (PRISMA-ScR).

Eligible studies included all forms of original empirical research evaluating interventions including: pilot studies; randomised control trials (RCTs); retrospective cohort studies reporting on THP in pregnant women and new mothers in low-and middle-income countries (LMICs). The Web of Science database search was limited to THP-specific studies published between 2008 and 2023 in English. Key data extracted included study design, population, setting, delivery mode, helper characteristics and training, safety, acceptability, feasibility, scalability, clinical and cost-effectiveness, and implementation barriers and facilitators.

**Results:**

Twelve studies evaluating THP were included, published between 2008 and 2023, conducted in six LMICs. Sample sizes ranged from 38 to 1,731 participants, with most studies conducted in rural or semi-urban settings. Eight studies (67%) were RCTs. The intervention was primarily delivered face-to-face, in ten studies. One study employed a remote/technology-assisted format, and another used a mixed delivery model combining face-to-face and digital methods.

Primary outcomes included depression severity measured using PHQ-9 (Patient Health Questionnaire-9) in nine studies, child development outcomes in two, delivery agent competency using the ENACT (Enhancing Assessment of Common Therapeutic Factors) rating in one, and cultural appropriateness in a qualitative study. Secondary outcomes measured included anxiety, disability and social support (five studies), cost-effectiveness (two studies), and child socio-emotional development (two studies).

**Conclusion:**

The THP has demonstrated effectiveness in reducing perinatal depression among women in low-resource settings. Its success across diverse contexts highlights its adaptability, cost-effectiveness and scalability. However, while THP improves maternal mental health, its impact on child developmental outcomes appears limited. Ongoing refinement is essential to broaden its impact on family wellbeing.

